# Pan-cancer analysis of Notch pathway-linked gene expression identifies tumour-specific and sex-stratified prognostic associations

**DOI:** 10.1016/j.ctro.2026.101268

**Published:** 2026-08-15

**Authors:** Helen O'Hea, V. Bourbonne, Laure Marignol

**Affiliations:** a Trinity St. James's Cancer Institute, Radiobiology and Molecular Oncology Research Group, Applied Radiation Therapy Trinity, Discipline of Radiation Therapy, Trinity College Dublin, Ireland; b Radiation Oncology Department, University Hospital Centre, Brest, France

**Keywords:** Notch, Cancer, Survival, Outcome, Biological sex

## Abstract

**Background:**

The Notch signalling pathway regulates cell fate, proliferation, and differentiation and consists of receptors (Notch1-4), ligands (JAG1-2, DLL1, DLL3-4), and downstream targets. Altered expression of Notch pathway-linked genes has been reported in multiple cancers, although their associations with survival may vary according to tumour type. Their relationship with biological sex and radiotherapy (RT) has been less extensively explored.

**Purpose:**

To explore associations between Notch pathway-linked gene expression and overall survival (OS) across multiple cancer types, and to examine whether these associations differ by biological sex or in an RT-treated lung cancer subgroup.

**Methodology:**

Gene expression and clinical data from patients across nine cancer types were analysed using publicly available datasets derived from The Cancer Genome Atlas (TCGA) and the Kaplan–Meier Plotter platform. Associations between the expression of 14 Notch pathway-linked genes and OS were evaluated using Kaplan–Meier survival analyses. Representative findings were further evaluated using multivariable Cox proportional hazards models incorporating clinical covariates and gene expression × sex interaction terms.

**Main findings:**

Across nine cancer types, 13 of the 14 Notch pathway-linked genes were significantly associated with OS in at least one tumour type. DLL3 and JAG1 demonstrated recurrent associations across multiple cancers, with higher DLL3 expression more frequently associated with poorer OS. Sex-stratified analyses identified additional prognostic associations; however, multivariable interaction analyses did not consistently support biological sex as an effect modifier. In the RT-treated lung adenocarcinoma subgroup, higher *JAG1* expression was associated with poorer OS, although this association was not confirmed in an adjusted pan-cancer RT validation cohort.

**Conclusion:**

This exploratory pan-cancer analysis identified associations between the expression of several Notch pathway-linked genes and OS, including recurrent associations involving *DLL3* and *JAG1*. Differences observed in sex-stratified analyses and the RT-treated lung adenocarcinoma subgroup warrant further investigation in independent cohorts.

## Highlights

• Systematic pan-cancer evaluation of 14 Notch pathway-linked genes across nine solid tumour types.
• Prognostic associations varied according to tumour type and following sex stratification.
• Multivariable validation did not consistently support biological sex as an effect modifier.
• DLL3 and JAG1 emerged as priority candidates for future translational investigation.

## Introduction

Notch signalling is emerging as one of the molecular pathways implicated in radioresistance, owing to its role in cancer stem cell maintenance, hypoxia, DNA damage responses and tumour cell survival [1], [2], [3].

Altered expressions of Notch signalling components have been observed in various malignancies [4], [5], functioning as either an oncogene or a tumour suppressor depending on the tissue and/or the cellular context [5], [6].

Notch signalling plays a role in the formation and maintenance of cancer stem cells (CSCs), a subpopulation of tumour cells with enhanced self-renewal capacity, enhanced differentiation and the ability to sustain tumour growth [7]. CSCs are resistant to radiation therapy (RT) due to their advanced DNA damage repair, cell-cycle redistribution, and reduced reactive oxygen species (ROS) levels, all of which contribute to treatment failure and recurrence [8]. Preclinical studies have demonstrated that ionising radiation can activate Notch signalling, promoting CSC survival and maintenance of stemness, while pharmacological inhibition of the pathway enhances tumour radiosensitivity in several tumour models, including glioblastoma and adenoid cystic carcinoma. [9], [10]

Targeting the Notch signalling pathway has emerged as a potential therapeutic strategy, with multiple approaches under investigation, including γ-secretase inhibitors, ADAM inhibitors, antibodies targeting specific Notch receptors/ligands, and inhibitors of the Notch transcription complex [4]. While early clinical studies have demonstrated antitumour effects, challenges remain in achieving adequate specificity and minimising toxicity to an acceptable level, as well as in addressing the complex regulatory needs of different cancer types, reflecting the highly context-dependent nature of Notch signalling [4].

Biological sex, distinct from socially constructed gender, may influence Notch signalling in cancer and is increasingly recognised as an important biological variable in cancer research [11], [12]. Beyond the effects of gonadal hormones, genetic and molecular mechanisms contribute to well-established sex differences in cancer incidence, survival, treatment toxicity, radiosensitivity and clinical outcomes [13], [14], [15], [16], [17], [18], [19]. As key cancer processes, including p53 signalling, angiogenesis and immune responses, exhibit sex differences [20], it is plausible that Notch signalling also contributes to these differences [21], [22], [23]. However, the influence of biological sex on Notch pathway activity and its association with clinical outcomes remain poorly understood.

This study systematically evaluated associations between Notch pathway-linked gene expression and overall survival across multiple cancer types, including exploratory radiotherapy and sex-stratified analyses to prioritise candidate biomarkers for future validation.

## Materials and methods

### Study design and patient cohorts

Gene expression and clinical data were obtained from the Kaplan–Meier Plotter platform [24],which integrates publicly available datasets from the Gene Expression Omnibus (GEO), European Genome-phenome Archive (EGA), and The Cancer Genome Atlas (TCGA). Overall survival (OS) was analysed across nine cancer types: bladder carcinoma, head and neck squamous cell carcinoma (HNSCC), kidney clear cell renal cell carcinoma (ccRCC), kidney papillary renal cell carcinoma (pRCC), lung adenocarcinoma, lung squamous cell carcinoma, oesophageal adenocarcinoma, oesophageal squamous cell carcinoma and rectal adenocarcinoma. Analyses were performed in the mixed population and stratified by recorded sex. Radiotherapy (RT)-specific analyses were restricted to lung adenocarcinoma because treatment data were available only for this cohort. A separate pan-cancer cohort of RT-treated patients was reconstructed from the TCGA database to evaluate the independent association between *JAG1* expression and overall survival following adjustment for available clinical covariates (Supplementary Table S1).

### Gene selection

Fourteen Notch pathway-linked genes were selected from the Kyoto Encyclopaedia of Genes and Genomes (KEGG) database, including receptors (*NOTCH1–4*), ligands (*JAG1, JAG2, DLL1, DLL3* and *DLL4*) and downstream targets (*HES1, HES5, HEY1, HEY2* and *HEYL*).

### Survival analyses

Kaplan–Meier survival analyses were performed using the KM Plotter platform. Patients were stratified into high- and low-expression groups using the platform's auto-selected best cut-off, with follow-up limited to 60 months. Hazard ratios (HRs), 95% confidence intervals (CIs), log-rank *p*-values and false discovery rates (FDRs) were recorded. Female oesophageal adenocarcinoma (*n* = 11) and oesophageal squamous cell carcinoma (*n* = 12) cohorts were excluded because of insufficient sample size. Sensitivity analyses using predefined median and quartile cut-offs were performed for representative findings.

### Multivariable validation

Representative sex-specific associations were evaluated using multivariable Cox proportional hazards models reconstructed from the underlying TCGA datasets. Models included age, recorded sex, tumour stage and grade where available, together with gene expression × sex interaction terms. An independent pan-cancer RT-treated TCGA cohort was similarly analysed to evaluate the association between JAG1 expression and OS after adjustment for available clinical covariates.

### Statistical analysis

Gene–cancer pairs with a log-rank *p*-value <0.05 and FDR ≤5% were considered statistically significant. Multivariable Cox regression results are reported as adjusted HRs with 95% CIs, with statistical significance defined as two-sided *p* < 0.05.

Additional methodological details are provided in the Supplementary Methods.

## Results

### Associations between Notch pathway-linked genes and overall survival in mixed population

**Fig. 1:**
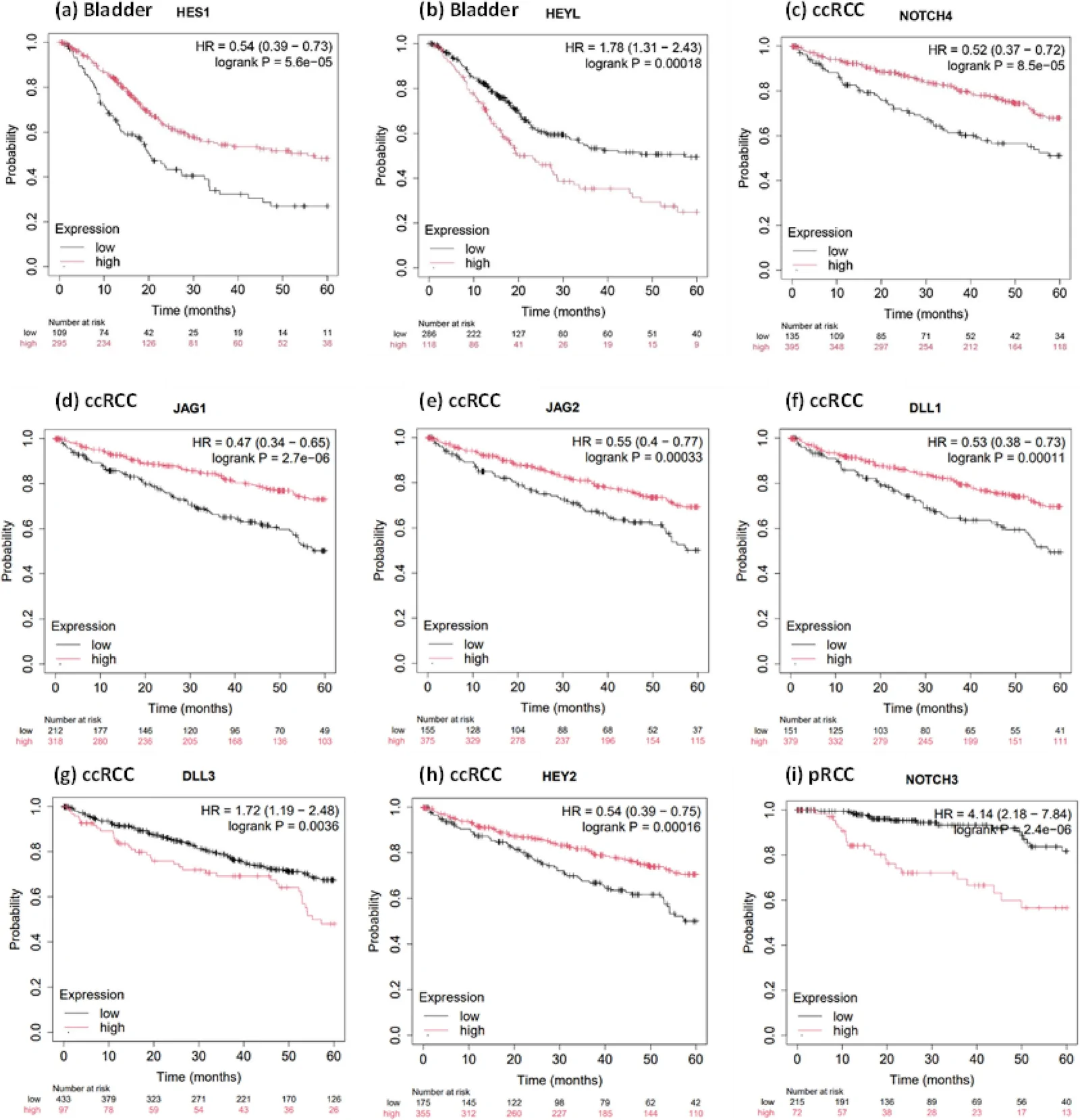

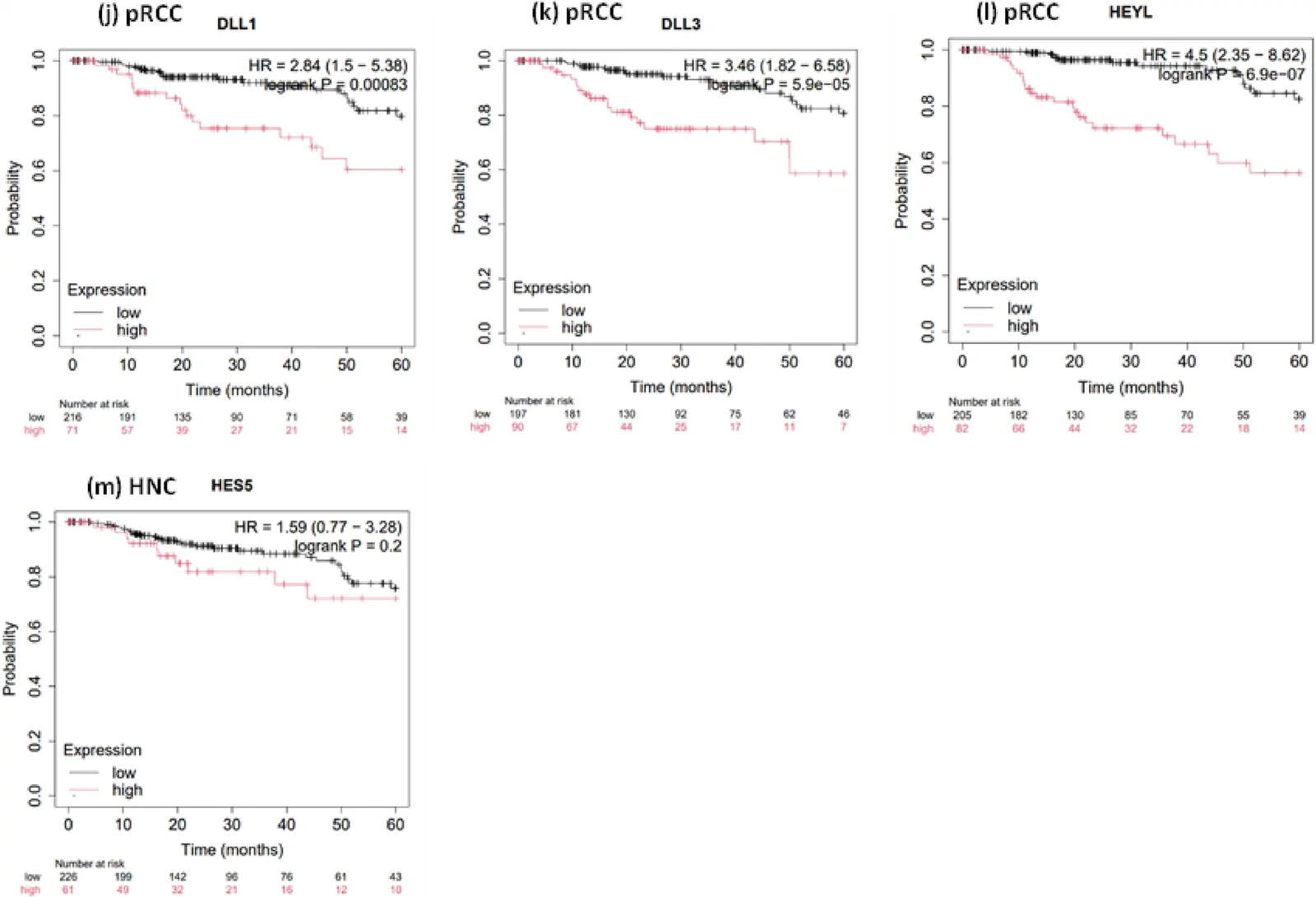
Kaplan–Meier curves showing the association between Notch pathway-linked gene expression and 60-month overall survival (OS) in the mixed population. (a) HES1–bladder carcinoma; (b) HEYL–bladder carcinoma; (c) NOTCH4–kidney clear cell renal cell carcinoma (ccRCC); (d) JAG1–ccRCC; (e) JAG2–ccRCC; (f) DLL1–ccRCC; (g) DLL3–ccRCC; (h) HEY2–ccRCC; (i) NOTCH3–kidney papillary renal cell carcinoma (pRCC); (j) DLL1–pRCC; (k) DLL3–pRCC; (l) HEYL–pRCC; and (m) HES5–head and neck squamous cell carcinoma (HNSCC). The y-axis represents the probability of overall survival and the x-axis represents follow-up time (months). High gene expression is shown in red and low gene expression in black. Numbers at risk are shown below each plot. Significant associations were defined as *p* < 0.05 and false discovery rate (FDR) ≤5%. (For interpretation of the references to colour in this figure legend, the reader is referred to the web version of this article.)

**Fig. 2:**
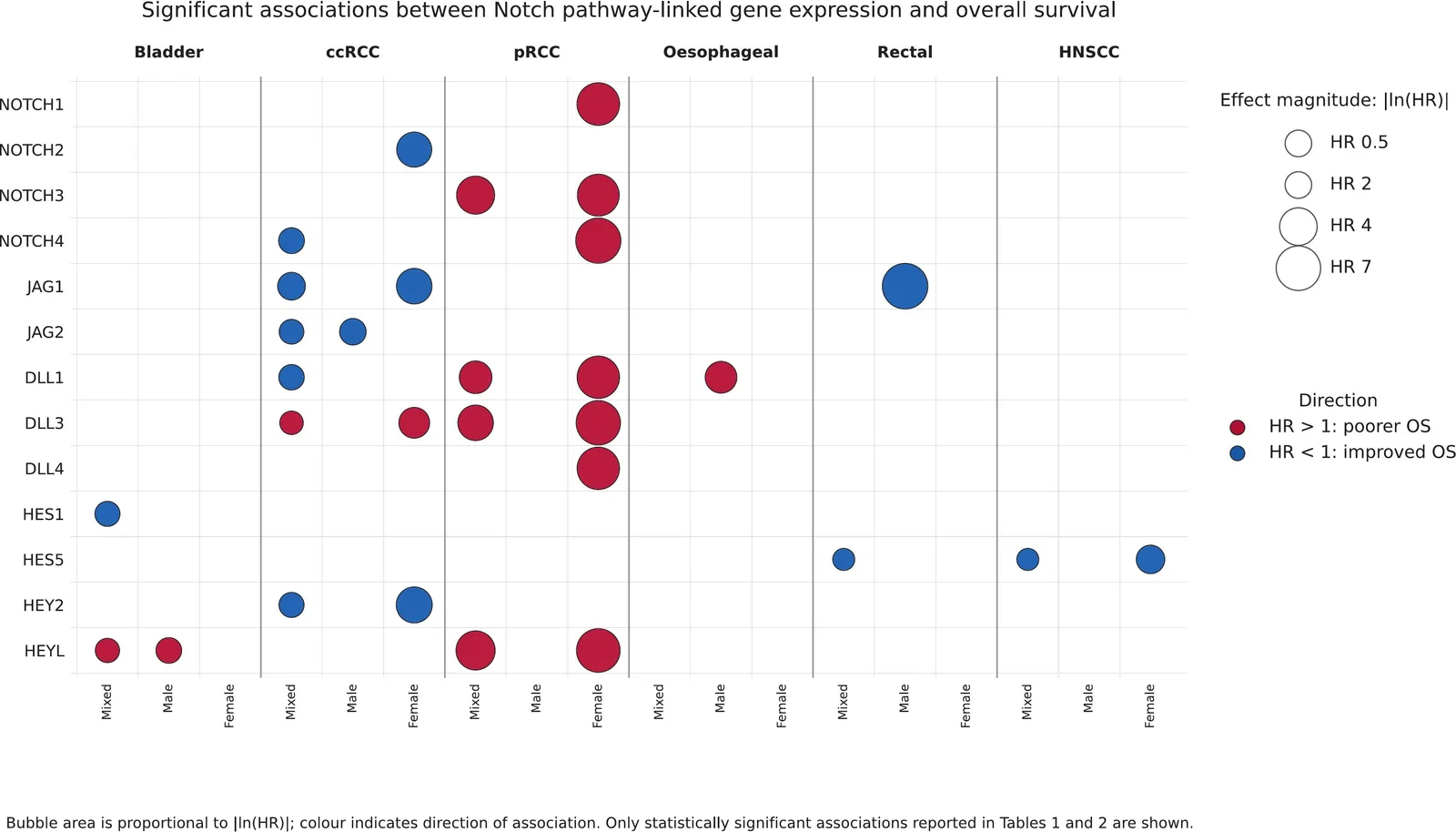
Significant associations between Notch pathway-linked gene expression and overall survival across cancer types. Bubble matrix summarising statistically significant associations identified in the mixed population and following stratification by biological sex. Rows represent Notch pathway-linked genes and columns represent cancer types, subdivided into mixed, male and female cohorts. Bubble colour indicates the direction of association, with red representing higher gene expression associated with poorer overall survival (HR >1) and blue representing higher gene expression associated with improved overall survival (HR <1). Bubble area is proportional to the magnitude of the association (|ln(HR)|). Only statistically significant associations (p<0.05 and FDR ≤5%) are shown; blank cells indicate that no significant association was identified. ccRCC, clear cell renal cell carcinoma; pRCC, papillary renal cell carcinoma; HNSCC, head and neck squamous cell carcinoma; HR, hazard ratio; FDR, false discovery rate.

The first analysis included all selected cancers in the mixed population. A total of 126 gene-cancer pairs were assessed, of which 13 showed a significant association with 5-year OS (*p* < 0.05, FDR ≤ 5%).(Table 1, Fig. 1). Of the 13 significant gene-cancer associations, seven were associated with improved OS in patients with high gene expression. These included *HES1* in bladder carcinoma (HR = 0.54), *Notch4, JAG1, JAG2, DLL1* AND *HEY2* in kidney ccRCC (HR = 0.47–0.55) and *HES5* in HNC SCC (HR = 0.62). In these gene-cancer pairs, higher expression was associated with improved OS. In contrast, 6 gene-cancer pairs showed that high gene expression was associated with poorer OS. These included *HEYL* in bladder carcinoma (HR = 1.78), *DLL3* in kidney ccRCC (HR = 1.72) and *Notch3, DLL1, DLL3*, and *HEYL* in kidney pRCC (HR = 2.84–4.50). In these gene-cancer pairs, higher expression was associated with poorer OS. (See Fig. 2.)

**Table 1:** Statistically significant gene-cancer pairs associated with OS in the mixed population.

Cancer	Gene	n	p-value	Auto cut-off HR(CI)	FDR(%)
Bladder	HES1	404	5.6e^−5^	0.54(0.39–0.73)	1
	HEYL	404	0.0002	1.78(1.31–2.43)	1
Kidney Renal Clear Cell	NOTCH4	530	8.5e^−5^	0.52(0.37–0.72)	1
	JAG1	530	2.7e^−6^	0.47(0.34–0.65)	1
	JAG2	530	0.0003	0.55(0.4–0.77)	3
	DLL1	530	0.0001	0.53(0.38–0.73)	1
	DLL3	530	0.0036	1.72(1.19–2.48)	1
	HEY2	530	0.0002	0.54(0.39–0.75)	1
Kidney Renal Papillary Cell	NOTCH3	287	2.4e^−6^	4.14(2.18–7.84)	1
	DLL1	287	0.0008	2.84(1.5–5.38)	2
	DLL3	287	5.9e^−5^	3.46(1.82–6.58)	1
	HEYL	287	6.9e^−7^	4.50(2.35–8.62)	1
Rectal Adenocarcinoma	HES5	499	0.0014	0.62(0.27–0.74)	3
HNC SCC	HES5	499	0.0014	0.62(0.27–0.74)	3

Across all significant gene-cancer pairs, Kaplan-Meier curves showed separation between the high- and low-expression groups. In kidney pRCC, *Notch3, DLL1, DLL3*, and *HEYL* showed an early and consistent divergence between expression groups over the 60-month follow-up period (Fig. 1). The observed variation in gene-cancer associations suggests that the relationship between Notch pathway gene expression and OS may differ according to tumour type. No significant gene-cancer associations were found in lung adenocarcinoma, lung SCC, oesophageal SCC and oesophageal adenocarcinoma. Sensitivity analyses using prespecified median and quartile expression cutoffs demonstrated that the principal renal cancer associations were generally robust to expression threshold selection (Supplementary Table S2).

### Associations between Notch pathway-linked genes and overall survival stratified by biological sex

In the sex-stratified analyses, each gene–cancer pair was evaluated separately in males and females. Female oesophageal adenocarcinoma (*n* = 11) and oesophageal squamous cell carcinoma (*n* = 12) cohorts were excluded because of insufficient sample size. The corresponding male oesophageal adenocarcinoma and squamous cell carcinoma cohorts (*n* = 69 each) were included, and sex-stratified analyses were successfully completed for all remaining cancer types.

In the male-only analysis, a total of 126 gene-cancer pairs were analysed. Five gene-cancer pairs, involving five unique genes, showed a statistically significant association with 5-year OS. Of these, 3 demonstrated that elevated expression was associated with improved OS: *HES1* in bladder carcinoma (HR = 0.53), *JAG2* in kidney ccRCC (HR = 0.50) and *JAG1* in rectal adenocarcinoma (HR = 0.13). Conversely, 2 gene-cancer pairs showed that high expression was linked to poorer OS, *HEYL* in bladder carcinoma (HR = 1.91) and *DLL1* in oesophageal adenocarcinoma (HR = 2.69) (Table 2)*.* None of the five gene–cancer associations that reached statistical significance in males also reached statistical significance in the corresponding female analyses (FDR ≥ 5%). Four of the five significant gene-cancer associations identified in the male analysis (*JAG1* in rectal adenocarcinoma, *JAG2* in kidney ccRCC, *HES1* in bladder carcinoma, and *HEYL* in bladder carcinoma) were also significant in the mixed-population analysis. In contrast, the association between *DLL1* expression and OS in oesophageal adenocarcinoma reached statistical significance only in the male subgroup. (Table 1, Table 2).

**Table 2:** Statistically significant gene-cancer pairs associated with OS, stratified by sex. n = number of patients, HR = hazard ratio, CI = 95% confidence interval, FDR = false discovery rate, M = male, F = female.

Cancer	Gene	Sex	n	p-value	Auto-cut off HR(CI)	FDR (%)
Bladder carcinoma	HEYL	M	298	0.0004	1.91(1.32–2.74)	3
Kidney Renal Clear Cell	NOTCH2	F	186	0.0080	0.30(0.14–0.63)	5
	JAG1	F	186	4.9e^−6^	0.29(0.16–0.51)	1
	JAG2	M	344	0.0007	0.5(0.33–0.75)	5
	DLL3	F	186	0.0016	2.54(1.4–4.65	2
	HEY2	F	186	0.0008	0.28(0.13–0.62)	5
Kidney Renal Papillary Cell	NOTCH1	F	76	0.0008	5.95(1.83–19.35	1
	NOTCH3	F	76	0.0013	5.59(1.71–18.2)	1
	NOTCH4	F	76	4.6e^−5^	7.39(2.4–22.79)	2
Kidney Renal Papillary Cell	DLL1	F	76	0.0010	5.84(1.79–19.09)	1
	DLL3	F	76	6.8e^−5^	6.88(2.29–20.66)	1
	DLL4	F	76	0.0005	5.84(1.89–18.05)	1
	HEYL	F	76	0.0003	6.48(2.07–20.27)	1
Oesophageal adenocarcinoma	DLL1	M	69	0.0039	2.69(1.34–5.41)	5
Rectal adenocarcinoma	JAG1	M	90	0.0002	0.13(0.04–0.45)	1
HNC SCC	HES5	F	133	0.0013	0.45(0.27–0.74)	3

In the female-only analysis, 98 gene-cancer pairs were analysed. Twelve gene-cancer pairs, involving 11 unique genes, showed a significant association with 5-year OS. Higher gene expression was associated with improved OS in four gene-cancer pairs: *NOTCH2*, *JAG1*, and *HEY2* in kidney ccRCC (HR = 0.28–0.30), and *HES5* in HNC SCC (HR = 0.45). Conversely, higher expression was associated with poorer OS in eight gene-cancer pairs: *DLL3* in kidney ccRCC, and *NOTCH1, NOTCH3, NOTCH4, DLL1, DLL3, DLL4*, and *HEYL* in kidney pRCC (HR = 2.54–7.39). None of the five gene–cancer associations that reached statistical significance in females also reached statistical significance in the corresponding male analyses (FDR ≥ 5%). Of the 12 significant gene-cancer associations identified in females, 11 were also detected in the mixed-population analysis. The association between *NOTCH2* expression and OS in kidney ccRCC was observed only in the female-only analysis (Table 1, Table 2).

#### Multivariable assessment of sex–gene interactions

To determine whether the apparent differences observed in the sex-stratified analyses reflected true effect modification by sex, multivariable Cox proportional hazards models were performed for two representative gene–cancer pairs that showed associations only within one sex: *DLL1* in oesophageal adenocarcinoma and *NOTCH2* in kidney clear cell renal cell carcinoma (Supplementary Table S3).

Continuous DLL1 expression remained independently associated with poorer overall survival after adjustment (HR = 1.54, 95% CI 1.22–1.94, *p* < 0.001), but no significant gene expression × sex interaction was observed. Likewise, continuous *NOTCH2* expression was not independently associated with overall survival or modified by sex. In contrast, dichotomised *NOTCH2* analyses identified a significant interaction with sex that persisted after adjustment, whereas *DLL1* results were unchanged.

### Radiation therapy, overall survival and Notch pathway-linked gene expression

Among the 14 gene–cancer pairs analysed in the RT-treated lung adenocarcinoma subgroup, only *JAG1* was significantly associated with 5-year overall survival, with higher expression associated with poorer outcome (HR = 2.3; Table 3). This association was not observed in the corresponding mixed-population analysis or confirmed in an adjusted RT-treated pan-cancer cohort, where *JAG1* expression was not independently associated with overall survival and no significant gene expression × sex interaction was identified (Supplementary Table S3).

**Table 3:** Statistically significant gene expression related to OS in the radiation therapy subgroup.

Cancer	Treatment	Gene	p-value	HR(CI)	FDR%	n
Lung A	RT	JAG1	0.0131	2.3(1.14–4.53)	5	65

HR = hazard ratio, CI = confidence interval, FDR = false discovery rate, n = number of patients, lung A = lung adenocarcinoma.

## Discussion

This study systematically evaluated associations between Notch pathway-linked gene expression and overall survival across multiple solid tumours. *DLL1*, *DLL3*, HEYL and JAG1 emerged as candidate genes for further biological investigation based on recurrent associations with overall survival. Given the increasing clinical interest in Notch-targeted therapies, particularly DLL3-directed agents [3], [25], [26], [27], these findings may help prioritise candidate biomarkers for future mechanistic and translational studies. Sex-stratified analyses identified several associations that were observed only in males or females, highlighting the potential value of considering biological sex during biomarker discovery [12]. However, formal multivariable Cox regression incorporating gene expression × sex interaction terms did not consistently support biological sex as an effect modifier.

Sex stratification identified prognostic associations that were not detected in the mixed-population analysis, including DLL1 in male oesophageal adenocarcinoma and NOTCH2 in female kidney ccRCC**.** *DLL1* is a ligand that regulates cell fate and differentiation [28]. Accumulating evidence supports its oncogenic role in multiple cancers [29]. Consistent with our findings, high DLL1 expression has been associated with poorer OS in oesophageal adenocarcinoma at both the protein and transcript levels, potentially through activation of the APE1–NF-κB axis and enhanced Notch signalling [30]. Similarly, elevated DLL1 protein expression has been reported in renal cancer stem cells, supporting a role in tumour progression [29]. Conversely, DLL1 has also been reported to exert tumour-suppressive effects by inhibiting tumour neovascularisation and enhancing anti-tumour immune responses [29]. In lung cancer, DLL1 overexpression suppresses JAG1–Notch signalling, promotes T-cell infiltration, and has been associated with improved responses to chemotherapy [29], [31], [32]. Together, these findings support a context-dependent role for DLL1 that may explain the opposing survival associations observed across tumour types in the present study.

Our analysis identified high DLL3 expression as being associated with poorer OS in females with kidney ccRCC and pRCC. *DLL3* is an atypical Notch ligand that inhibits Notch signalling and prevents Notch receptor activity. It is normally expressed at low levels in healthy tissues but is present on the surface of specific tumour cells [29]. Although evidence for *DLL3's* specific role in kidney RCC is limited [33], high expression has been associated with poorer prognosis in several cancers [29]. In small-cell lung cancer, DLL3 promotes tumour growth, migration and invasion through modulation of SNAI1/Snail [34], [35], while in invasive breast cancer it is associated with poorer prognosis and altered immune infiltration, potentially through cell-cycle regulation and inhibition of apoptosis [3], [36]. These findings are consistent with the poorer OS observed in study. However, because our analyses were based on bulk tumour transcriptomic data, these associations should be interpreted as prognostic rather than mechanistic. Further studies are required to clarify the biological significance of DLL3 expression in kidney RCC before its potential as a prognostic biomarker can be established.

*HEYL* emerged as a significant gene in this study, with high expression associated with poorer OS in kidney pRCC and bladder carcinoma. These associations were observed following sex stratification, being detected in males in bladder carcinoma and in females in kidney pRCC. *HEYL* is a downstream transcriptional target of Notch signalling and regulates proliferation, differentiation, and cell fate decisions [37], [38]. *HEYL* has demonstrated tumour-suppressive roles in specific contexts, including limiting metastases in colorectal cancer and promoting p53-mediated apoptosis in hepatocellular carcinoma [39], [40]. However, increasing evidence supports an oncogenic role for *HEYL* in cancer [41], [42]. In breast cancer, *HEYL* promotes angiogenesis in both tumour cells and the tumour-associated endothelium, and in oesophageal SCC it activates LAMA3 transcription, promoting EMT and reducing radiosensitivity [41], [42]. In bladder cancer, single-cell RNA sequencing analyses have demonstrated that high HEYL protein expression promotes tumour progression and chemoresistance through angiogenesis and EMT, aligning with the poorer OS detected in the present study [43]. Furthermore, siRNA-mediated knockdown of HEYL protein inhibited the proliferation and invasion of bladder cancer cells [44].

In kidney pRCC, evidence regarding the specific role of *HEYL* is limited. [33] However, CBX2-regulated ASPH has been proposed to promote carcinogenesis and unfavourable prognosis via Notch signalling [45]. Although HEYL overexpression may be consistent with enhanced Notch pathway-linked biology [46], our analysis cannot determine whether increased *HEYL* expression directly reflects pathway activation, as bulk mRNA expression captures contributions from both tumour cells and the surrounding tumour microenvironment. Notably, high expression of *Notch1, DLL4* and *HEYL* was associated with poorer OS. Further functional studies are therefore required to determine the biological significance of these associations before HEYL can be considered a reliable prognostic biomarker or therapeutic target.

High JAG1 expression was associated with improved OS in female kidney ccRCC and male rectal adenocarcinoma, contrasting with reports linking elevated JAG1 protein expression to poor prognosis in gastric, hepatocellular, breast and ovarian cancers [47], [48]. *JAG1* is a Notch ligand frequently implicated in tumour proliferation, angiogenesis, metastasis, CSC maintenance, and immune evasion [49]. In kidney ccRCC, high JAG1 protein expression in tumour samples has been associated with oncogenic properties, whereas analyses of TCGA mRNA data have suggested a protective association [50], [51]. Similarly, although JAG1 is widely regarded as oncogenic in colorectal cancer through promotion of EMT, metastasis and treatment resistance, our findings suggest that its prognostic significance may vary according to tumour context [52]. These conflicting findings likely reflect the context-dependent role of JAG1 and methodological differences between studies. As our analysis was based on bulk tumour mRNA expression, whereas most previous studies evaluated protein expression, direct comparisons should be interpreted cautiously because mRNA abundance does not necessarily reflect protein expression or Notch pathway activity [53].

Sex-stratified analyses identified several prognostic associations that were not detected in the mixed-population analysis. However, multivariable Cox regression incorporating gene expression × sex interaction terms did not consistently support biological sex as an effect modifier. Accordingly, these findings should be interpreted as sex-stratified prognostic associations rather than definitive sex-dependent biological effects. Nevertheless, they highlight the value of considering biological sex during biomarker discovery, as subgroup analyses may reveal clinically relevant associations warranting further investigation. Emerging evidence suggests that Notch signalling may contribute to sex differences in cancer [54], [55], consistent with established differences in cancer incidence, prognosis and radiosensitivity [14], [16]. Potential mechanisms include differences in DNA damage responses, immune regulation and genes escaping X-chromosome inactivation [11], [56]. Further studies in larger, clinically annotated cohorts are required to determine whether these observations translate into genuine sex-dependent prognostic effects.

Exploratory analyses of RT-treated lung adenocarcinoma identified higher JAG1 expression as being associated with poorer overall survival. However, this association was not reproduced in an adjusted TCGA-derived pan-cancer RT cohort, where JAG1 expression was not independently associated with overall survival and no significant JAG1 × sex interaction was observed. These findings should therefore be considered hypothesis-generating and do not support a role for JAG1 as an independent biomarker of radiotherapy response. Nevertheless, experimental studies support a biologically plausible role for JAG1 in radiotherapy response. High JAG1 expression has been associated with tumour progression, poor prognosis and maintenance of radioresistant lung cancer stem cells through enhanced DNA repair and reduced reactive oxygen species [8], [57], [58], [59]. Furthermore, inhibition of JAG1 by miR-153 has been shown to impair cancer stem cell function and enhance radiosensitivity in preclinical models [59], [60], [61]. Although these findings provide biological support for JAG1 as a candidate radiotherapy biomarker, larger disease-specific cohorts are required to determine its clinical relevance.

This study has several limitations. The analyses were retrospective and based on publicly available transcriptomic datasets, requiring validation in independent cohorts. Gene expression was assessed using bulk tumour mRNA, which does not directly reflect protein expression or Notch pathway activity. Comprehensive multivariable validation across all gene–cancer pairs was beyond the scope of this pathway-level discovery study, and the exploratory radiotherapy findings require confirmation in larger disease-specific cohorts.

## Conclusion

This study provides a systematic pathway-level evaluation of associations between Notch pathway-linked gene expression and overall survival across multiple solid tumours. The findings identify candidate prognostic genes whose associations vary according to tumour type and highlight the importance of considering biological sex during biomarker discovery. These observations are hypothesis-generating and provide a framework for prioritising Notch pathway-linked genes for future mechanistic studies and independent clinical validation.

## CRediT authorship contribution statement

**Helen O'Hea:** Writing – review & editing, Writing – original draft, Methodology, Formal analysis, Data curation. **V. Bourbonne:** Writing – review & editing, Validation, Methodology, Formal analysis, Data curation. **Laure Marignol:** Writing – review & editing, Writing – original draft, Visualization, Supervision, Project administration, Methodology, Investigation, Data curation, Conceptualization.

## Declaration of competing interest

Prof. Laure Marignol is an editorial board member for CTRO. Prof. Laure Marignol was not involved in the peer-review of this article and had no access to information regarding its peer-review. Full responsibility for the editorial process for this article was delegated to another journal editor.

## Appendix A Supplementary data

Supplementary Table S1. Supplementary material 1 Baseline characteristics of the reconstructed validation cohorts. Clinical and demographic characteristics of the DLL1 oesophageal adenocarcinoma, NOTCH2 kidney clear cell renal cell carcinoma (ccRCC), and JAG1 radiotherapy-treated pan-cancer cohorts used for multivariable survival analyses. Values are presented as median [interquartile range], mean (standard deviation), or number (%), as appropriate. Cohort sizes reflect patients with complete data for the covariates included in the corresponding multivariable models. RT, radiotherapy; IQR, interquartile range; SD, standard deviation; GX, grade not assigned.

Supplementary material 2 Supplementary Table S2. Sensitivity analysis of selected Notch pathway gene–cancer associations using prespecified expression cut-offs. Associations between gene expression and overall survival were re-evaluated using median, lower-quartile and upper-quartile expression cut-offs. Associations were classified as retained when statistical significance was maintained at the specified cut-off and attenuated when the association was no longer statistically significant. HR, hazard ratio; CI, confidence interval.

Supplementary material 3 Supplementary Table S3. Multivariable Cox proportional hazards analyses of representative associations identified in the Kaplan–Meier discovery analyses. Associations between gene expression and overall survival were evaluated using continuous-expression models and dichotomised models based on the original KM Plotter cut-offs, with adjustment for available clinical covariates. Continuous-expression models were considered the primary analyses, while dichotomised models were included to facilitate comparison with the discovery analyses. Gene expression × sex interaction terms were additionally evaluated where applicable. HR, hazard ratio; CI, confidence interval; RT, radiotherapy.
