# Supplementary material for: Pan-cancer analysis of Notch pathway-linked gene expression identifies tumour-specific and sex-stratified prognostic associations
Source: Clin Transl Radiat Oncol. 2026 Aug 15;61:101268. doi: 10.1016/j.ctro.2026.101268 (PMC13524761; doi:10.1016/j.ctro.2026.101268)
Supplement: Supplementary file 1 [file mmc1.docx]

## Supplementary Table S1. Baseline characteristics of the reconstructed validation cohorts.

Values are presented as median [interquartile range], mean (standard deviation), or number (%), as appropriate.

| Characteristic | DLL1 cohort (Oesophageal adenocarcinoma) | NOTCH2 cohort (Kidney ccRCC) | JAG1 RT cohort (Pan-cancer) |
| --- | --- | --- | --- |
| Number of patients, n | 71 | 494 | 1826 |
| Tumor type(s), n (%) | ESCA: 71 (100.0%) | KIRC: 494 (100.0%) | BRCA: 520 (28.5%); HNSC: 274 (15.0%); THCA: 274 (15.0%); UCEC: 200 (11.0%); CESC: 142 (7.8%); Other: 416 (22.8%) |
| Age at initial pathologic diagnosis, years, median [IQR] | 70.0 [58.0–77.0] (n=71) | 61.0 [52.0–70.0] (n=494) | 58.0 [47.0–66.0] (n=1826) |
| Age at initial pathologic diagnosis, years, mean (SD) | 66.5 (12.2) (n=71) | 60.7 (12.1) (n=494) | 56.5 (13.9) (n=1826) |
| Sex, n (%) | FEMALE: 10 (14.1%); MALE: 61 (85.9%) | FEMALE: 177 (35.8%); MALE: 317 (64.2%) | FEMALE: 1257 (68.8%); MALE: 569 (31.2%) |
| Tumor stage, n (%) | Stage I: 9 (12.7%); Stage II: 18 (25.4%); Stage III: 25 (35.2%); Stage IV: 5 (7.0%); Missing/Not available: 14 (19.7%) | Stage I: 241 (48.8%); Stage II: 54 (10.9%); Stage III: 121 (24.5%); Stage IV: 75 (15.2%); Missing/Not available: 3 (0.6%) | Stage 0: 13 (0.7%); Stage I: 282 (15.4%); Stage II: 420 (23.0%); Stage III: 358 (19.6%); Stage IV: 240 (13.1%); Missing/Not available: 513 (28.1%) |
| Histological grade, n (%) | G1: 1 (1.4%); G2: 23 (32.4%); G3: 24 (33.8%); GX: 23 (32.4%) | G1: 13 (2.6%); G2: 207 (41.9%); G3: 199 (40.3%); G4: 67 (13.6%); GX: 5 (1.0%); Missing/Not available: 3 (0.6%) | G1: 65 (3.6%); G2: 327 (17.9%); G3: 321 (17.6%); G4: 4 (0.2%); GX: 30 (1.6%); High Grade: 16 (0.9%); Low Grade: 6 (0.3%); Missing/Not available: 1057 (57.9%) |
| Tumor purity, median [IQR] (RT/JAG1 cohort only) | 0.61 [0.45–0.72] (n=71) | 0.56 [0.44–0.65] (n=494) | 0.62 [0.46–0.76] (n=1826) |
| Deaths, n (%) | 34 (47.9%) | 166 (33.6%) | 344 (18.8%) |

Abbreviations: RT, radiotherapy; IQR, interquartile range; SD, standard deviation; GX, grade not assigned. Cohort sizes differ from the corresponding KM Plotter analyses because only patients with complete data for the covariates included in the multivariable models (including tumour purity where applicable) were retained.
