# Supplementary material for: Pan-cancer analysis of Notch pathway-linked gene expression identifies tumour-specific and sex-stratified prognostic associations
Source: Clin Transl Radiat Oncol. 2026 Aug 15;61:101268. doi: 10.1016/j.ctro.2026.101268 (PMC13524761; doi:10.1016/j.ctro.2026.101268)
Supplement: Supplementary file 2 [file mmc2.docx]

**Supplementary Table S2. Sensitivity analysis of selected Notch pathway gene–cancer associations using prespecified expression cutoffs.**

| Gene | Cancer | Cut-off | HR (95% CI) | P value | Association across prespecified cutoffs |
| --- | --- | --- | --- | --- | --- |
| DLL3 | Kidney renal clear cell carcinoma | Median | 1.64 (1.20–2.24) | p=0.0015 | Retained |
|  |  | Lower quartile | 1.4 (1-1.95) | p=0.047 | Retained |
|  |  | Higher quartile | 1.72 (1.19-2.48) | p=0.0036 | Retained |
|  | Kidney renal papillary cell carcinoma | Median | 1.84 (1.01-3.36) | p=0.044 | Retained |
|  |  | Lower quartile | 2.59 (1.08-6.19) | p=0.027 | Retained |
|  |  | Higher quartile | 3.16 (1.65-6.04) | p=0.0024 | Retained |
| NOTCH3 | Kidney renal clear cell carcinoma | Median | 0.72 (0.52-1) | P=0.052 | Attenuated |
|  |  | Lower quartile | 1.13 (0.76-1.66) | P= 0.55 | Attenuated |
|  |  | Higher quartile | 0.77 (0.51-1.14) | P=0.19 | Attenuated |
|  | Kidney renal papillary cell carcinoma | Median | 2.44 (1.23-4.83) | P=0.0084 | Retained |
|  |  | Lower quartile | 2.14 (0.89-5.11) | P=0.811 | Attenuated |
|  |  | Higher quartile | 4.14 (2.18-7.84) | P= 2.4e-6 | Retained |
| JAG1 | Kidney renal clear cell carcinoma | Median | 0.56 (0.4-0.78) | P=0.00049 | Retained |
|  |  | Lower quartile | 0.5 (0.36-0.7) | P=3.4e-05 | Retained |
|  |  | Higher quartile | 0.62 (0.41-0.96) | P=0.03 | Retained |
|  | Kidney renal papillary cell carcinoma | Median | 1.03 (0.54-1.94) | P=0.93 | Attenuated |
|  |  | Lower quartile | 3.87 (1.19-12.6) | P=0.015 | Retained |
|  |  | Higher quartile | 1.17 (0.57-2.41) | P=0.67 | Attenuated |
