# Supplementary material for: Pan-cancer analysis of Notch pathway-linked gene expression identifies tumour-specific and sex-stratified prognostic associations
Source: Clin Transl Radiat Oncol. 2026 Aug 15;61:101268. doi: 10.1016/j.ctro.2026.101268 (PMC13524761; doi:10.1016/j.ctro.2026.101268)
Supplement: Supplementary file 3 [file mmc3.docx]

# Supplementary Table S3

**Multivariable Cox proportional hazards analyses evaluating representative findings from the Kaplan–Meier discovery analyses.**

| **Gene** | **Cancer type (N= total patient number)** | **Patients included in the analysis (number of events)** | **Expression model** | **Adjusted for** | **Gene HR (95% CI)** | **Gene p-value** | **Interaction p-value** |
| --- | --- | --- | --- | --- | --- | --- | --- |
| DLL1 | Oesophageal adenocarcinoma | 80 (34) | Continuous | Age, sex, stage, grade | 1.54 (1.22–1.94) | <0.001 | 0.747 |
|  |  |  | Dichotomised (KM Plotter) | Age, sex, stage, grade | 3.20 (1.40–7.32) | 0.006 | 0.251 |
| NOTCH2 | Kidney clear cell renal cell carcinoma | 530 (166) | Continuous | Age, sex, stage, grade | 1.06 (0.85–1.32) | 0.603 | 0.060 |
|  |  |  | Dichotomised (KM Plotter) | Age, sex, stage, grade | 0.84 (0.55–1.27) | 0.403 | 0.015 |
| JAG1 | RT-treated pan-cancer cohort | 2806 (343) | Continuous | Age, sex, histology, stage, grade, tumour purity* | 0.99 (0.89–1.11) | 0.919 | 0.285 |
|  |  |  | Dichotomised (KM Plotter) | Age, sex, histology, stage, grade, tumour purity* | 1.00 (0.90–1.12) | 0.981 | 0.370 |

**Abbreviations:** HR, hazard ratio; CI, confidence interval; RT, radiotherapy. Events refer to deaths observed during follow-up for overall survival analyses. *Covariates were included where available. Continuous-expression models were considered the primary analyses. Dichotomised models using the original KM Plotter cut-off were performed as secondary analyses to facilitate comparison with the discovery analyses. Formal gene expression × sex interaction terms were fitted for both continuous and dichotomised models where applicable.
